# The implementation of an integrated workplace health promotion program in Dutch organizations ‐ A mixed methods process evaluation

**DOI:** 10.1371/journal.pone.0308856

**Published:** 2024-11-01

**Authors:** Denise J. M. Smit, Sandra H. van Oostrom, Josephine A. Engels, Suzan Mooren-van der Meer, Karin I. Proper

**Affiliations:** 1 Centre for Prevention, Lifestyle and Health, National Institute for Public Health and the Environment, Bilthoven, The Netherlands; 2 Department of Public and Occupational Health, Amsterdam UMC, Vrije Universiteit Amsterdam, Amsterdam Public Health Research Institute, Amsterdam, The Netherlands; 3 Occupation and Health Research Group, HAN University of Applied Sciences, Nijmegen, The Netherlands; 4 Musculoskeletal Rehabilitation Research Group, HAN University of Applied Sciences, School for Allied Health, Nijmegen, The Netherlands; Bangladesh Open University, BANGLADESH

## Abstract

**Objective:**

To gain insight into 1) the degree of implementation of an integrated workplace health promotion program (WHPP) 2) the perceptions of employers and employees regarding an integrated WHPP and 3) the contextual factors that hindered or enhanced implementation.

**Methods:**

Data were collected by means of questionnaires, interviews among 19 employees, supervisors and HR-professionals, monitoring charts and observations at 6–10 months after the start of the implementation of the integrated WHPP. To evaluate the implementation process, ten process indicators from the evaluation frameworks of Nielsen & Randall and Wierenga were assessed. Descriptive analyses were performed for the process indicators as measured by questionnaires, monitoring charts and observations. Interviews with employers and employees were recorded, transcribed and then coded by two researchers independently by means of thematic coding.

**Results:**

The results cover the following topics: implemented activities, the working group, engagement of employees, the role of management and policy and organizational preconditions. Although the criteria of the WHPP were not completely met, various activities were implemented in all participating organizations. Working groups consisting of Human Resources professionals, supervisors and employees, who selected and implemented activities, were composed within each organization. 22% of the employees did not feel involved in the implementation process. The absence of organizational policies regarding WHP hampered implementation. Organizations had the intention to continue with the integrated WHPP, which requires sufficient time and budget.

**Conclusions:**

The implementation of the integrated WHPP appeared to be challenging and complex. Working groups indicated that they made the first important steps in integrating WHP in their organization and had the intention to continue with the implementation. However, to increase the impact, employers and employees should have the opportunity to implement and participate in WHP. Hence, organizational policies regarding WHP and active support of higher management are expected to be essential.

## Introduction

Workplace health promotion programs (WHPPs) have been studied extensively, and despite proven effectiveness, evidence remains limited [1–3]. Implementation of the WHPPs might play a significant role in this, as poor implementation, such as insufficient communication and adoption into practice can negatively impact effectiveness [4–8]. To improve implementation and thus effectiveness of future WHPPs, it is of importance to study not only the effectiveness of these programs, but also the implementation process [4]. First of all, process evaluations can provide insight in the factors contributing to the (lack of) effectiveness and the barriers and facilitators for implementation that play a role across different settings [4, 7, 9, 10]. Secondly, a deviation from the original implementation plan does not exclusively lead to smaller effects, but can also achieve positive results, which will only be known when the implementation process is properly evaluated [5]. Unfortunately, despite growing attention for the value of process evaluations, they are still being conducted sparsely [1, 4, 7, 10, 11]. Murta et al. (2007) reported that process evaluations concerning WHPPs with a focus on stress management were often incomplete, lacked a theoretical framework and were not planned prior to implementation [12]. This indicates that there is a need for systematically conducted, comprehensive process evaluations regarding WHPPs.

In this study the implementation process of an integrated WHPP to improve the overall lifestyle of employees was evaluated. In which lifestyle is defined as a combination of different health behaviors i.e. physical activity, nutrition, smoking, alcohol consumption and stress. The integrated WHPP of concern was built upon a European Good Practice, The Lombardy Workplace Health Promotion Network (LWHPN), and tailored to the Dutch context [13–15]. One of the key elements of the integrated WHPP is that it includes activities at both the individual and organizational level for multiple health behaviors, e.g. physical activity and nutrition. Another key element of the integrated WHPP is the selection and implementation of activities that fit the organization and the needs of employees by a working group, consisting of HR, supervisors and employees, which is composed within each organization. In addition the working group creates awareness, enthusiasm and support within the organization. To examine whether these elements were actually applied in practice, the degree of implementation should be evaluated [4, 5, 16]. For instance, the integrated WHPP may only yield a positive result when employees are aware of, participate in and actually receive the integrated WHPP as intended, i.e. including both key elements. Furthermore, to gain insight in the success or failure of the integrated WHPP, behaviors and perceptions of stakeholders should be assessed. The motivation, knowledge, skills and opportunities of those involved in the implementation affect the actual implementation of the integrated WHPP [17]. Furthermore, various contextual factors, i.e. organizational structure or culture and characteristics of stakeholders, might either hamper or support the implementation process [5, 18].

Hence, to illuminate the factors associated with success or failure of the integrated WHPP in practice, the aim of this study was to evaluate the implementation process of the integrated WHPP. Specifically, we provide insight in 1) the degree of implementation of the integrated WHPP, 2) the perceptions of stakeholders and 3) the contextual factors affecting the implementation of an integrated WHPP.

## Materials and methods

### Design

A mixed methods process evaluation was conducted alongside a two armed cluster randomized controlled trial (C-RCT) over a period of 6 to 10 months from baseline (between March 2022 and November 2023). For the C-RCT, randomization was carried out at the level of working locations, meaning that there was a control and intervention condition within each organization. Data were collected by means of an online questionnaire, interviews, monitoring charts and observations at the workplace.

The Medical Ethical Committee of the VU University Medical Center (VUmc, Amsterdam, the Netherlands) approved the study protocol (2021.0402). Written informed consent was obtained from all participants prior to commencing the study.

### Study population

The integrated WHPP was implemented in four Dutch organizations in different occupational sectors (i.e. two educational organizations, an assurance, tax and consulting organization, and a retail organization). More details about the organizations are provided in S1 Table. Organizations were recruited through the network of the project team members, co-workers and branch specific networks. Organizations could participate when they employed approximately 200 employees and did not yet implemented a WHPP comparable to the integrated WHPP, i.e. including activities on both the individual and organizational level for multiple health behaviors. Employees within these organizations were invited to participate in the study. Within the first educational organization, only the ICT- and facility department participated. Employees were recruited between January 14^th^ 2022 and March 29^th^ 2023. See for more details about the study population and study design, the protocol paper [15]. In the C-RCT 180 employees were included at baseline and those in the intervention condition (n = 90) received additional questions regarding the process evaluation in the questionnaire at six months. Interviews took place among a subgroup of the participants in the intervention condition. These employees were randomly selected using an online number generator, and received an e-mail from the researcher to invite them to participate in an interview. Moreover, one to three Human Resources (HR) professionals and/or supervisors, hereafter referred to as ‘employers’, from each organization, involved in the implementation of the integrated WHPP, were invited for an interview. Employers were recruited between December 14^th^ 2022 and September 26^th^ 2023

### The integrated WHPP

**Fig 1:**
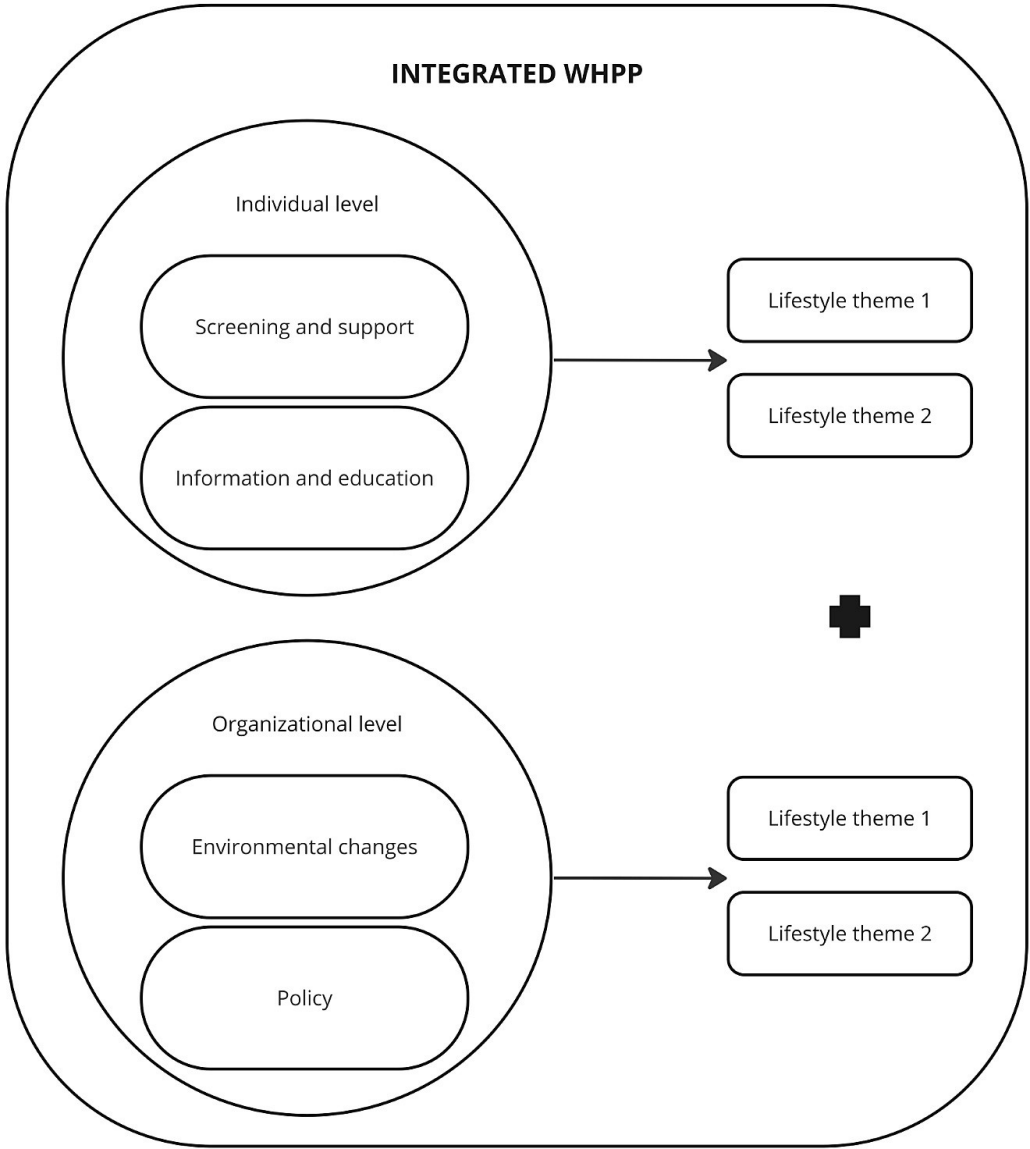
An overview of the integrated WHPP.

Contact persons within each organization received 1) a catalogue with a varying range health promoting activities on both the individual and organizational level and multiple health behaviors (i.e. physical activity, nutrition, mental balance, sleep, smoking and alcohol consumption), based on the CHRODIS+ toolkit [19] and 2) an implementation plan to support successful implementation [15]. As a first step, a working group consisting of HR professionals, employees and supervisors was composed within each organization. They selected and implemented activities in the intervention condition, based on needs and preferences of employees according to the criteria of the integrated WHPP. To meet the criteria of the integrated WHPP, they were asked to implement at least one activity at the individual and one activity at the organizational level, where both had to be performed for at least two health behaviors (Fig 1) within six months after the start of the implementation [15]. This means that in total four activities had to be implemented by each organization.

### Implementation components

Three components of the implementation process, i.e. degree of implementation, perceptions of employees and employers and contextual factors, were assessed by ten process indicators from the evaluation frameworks of Nielsen & Randall and Wierenga [1, 20]. Table 1 provides a detailed description of the implementation components and indicators, their definition and how and when they were measured. The first component included in this study is the degree of implementation, which comprises the following process indicators: implementation strategy [1], fidelity, dose delivered, dose received, recruitment and reach [20]. Secondly, the perceptions of employees and employers were assessed following the process indicators: satisfaction [20] and participants’ mental models [1]. The third and last component, contextual factors, consists of context and maintenance indicators [20].

**Table 1:** Overview of implementation process indicators that were measured among employees, supervisors or HR professionals or were observed at the workplace including the definition and the timing of the measurement from baseline.

Main implementation process component	Process indicator	Definition	Employees		Supervisor/HR		Workplace	
			*Questionnaire*	*Interviews*	*Interviews*	*Monitoring charts*	*Observations*	
			6 months	7–10 months	7–10 months	6 and 8–10 months	Baseline	7–10 months
Degree of implementation	Implementation strategy	*Roles and behaviors of key stakeholders*, *i*.*e*. *members of the working group*			X	X		
	Fidelity	*Compliance to the integrated WHPP and use of the catalogue and* *implementation plan*			X	X		
	Dose delivered	*Number and type of activities implemented by the employer*				X	X	X
	Dose received	*Proportion of employees that received the integrated WHPP*	X	X		X		
	Recruitment of employees	*Sources and procedures used to stimulate participation of employees in activities*	X	X		X	X	X
	Reach	*Proportion of employees who were aware of the implemented activities*	X	X				
Perceptions of employees and employers	Satisfaction	*Opinion and satisfaction of employees and employers about the implemented activities and its implementation*	X	X	X			
	Participants’ mental models	*Perceptions and appraisals of employees and employers about the integrated WHPP*	X	X	X			
Contextual factors	Context	*Barriers and facilitators perceived by employees and employers for participation and implementation respectively*	X	X	X			
	Maintenance	*Prerequisites for continuation of the integrated WHPP according to employers*			X			

Regarding the contextual factors, the Consolidated Framework for Implementation Research (CFIR) was applied to analyze and report the data [21]. The CFIR is a framework applied in implementation research and consists of five domains: 1) innovation, 2) outer setting, i.e. the external environment, 3) inner setting, i.e. the working environment, 4) individuals and 5) process. The Capability, Opportunity, Motivation, Behavior (COM-B) model, is integrated in the individuals domain of the CFIR [21, 22]. COM-B can help to describe behavior of individuals by means of the interaction between capability (psychological or physical), opportunity (social or physical) and motivation (reflective or automatic) [22].

#### Data collection

Online questionnaires were completed by employees at six months follow up. Questionnaires were developed based on the process indicators (dose received, recruitment, reach, satisfaction, mental models and context) and included questions about 1) perceived involvement of employees in selection and implementation of activities and if implemented activities met their needs, 2) perceived support and commitment of supervisors during the program, 3) satisfaction and awareness about implemented activities and communication channels used by implementers, 4) the extent to which employees participated in activities and 5) barriers and facilitators for participation, based on the COM-B model (physical and psychological capability, physical and social opportunity, automatic and reflective motivation). The answer categories, a five-point Likert scale ranging from ‘totally disagree’ to ‘totally agree’, were in line with those used in previous process evaluations or other studies [23, 24].

Around 8–10 months after the start of the implementation of the integrated WHPP, semi-structured interviews were conducted with employees (both employees who were part of the working group and employees who were not) and employers i.e. HR professionals and supervisors who were part of the working group. A preliminary plan about the sample size of interviewees included to interview two employees, one employee in the working group and two employers. However, deviations of this plan could occur and depended on for instance the size of the organization, willingness to participate and data saturation. The interview guideline for employees consisted of questions about 1) if employees participated in activities or not and for what reason, 2) which communication channels were used according to employees, whether they thought these channels were suitable and if they appreciated the use of these channels, 3) their attitude towards and believe in the integrated WHPP, and 4) their satisfaction about the integrated WHPP. The employer interview guideline included questions regarding 1) the roles and behaviors of implementers, 2) their attitude towards and believe in the integrated WHPP, 3) use of the program materials, i.e. the catalogue and implementation plan, 4) perceived barriers and facilitators for implementation, and 5) satisfaction and motivation to continue with the integrated WHPP. Both guidelines were developed based on process indicators as depicted in Table 1. Interviews were conducted by DS. If DS was familiar with the interviewee, another researcher conducted the interview, to mitigate potential bias.

A monitoring chart was completed by one member of each working group from the start of the implementation of the integrated WHPP (S2 Table). In these monitoring charts the implemented activities, the amount of time necessary to implement activities, timing of implementation and the applied communication strategies to inform employees about activities were registered.

Observations were done per location for each organization. For one organization a sample of the locations was visited, due to the large number of separate locations throughout the country. One of the researchers conducted the observations by means of a checklist to assess 1) adjustments to the physical working environment and 2) visible communication concerning adjustments or activities, i.e. posters and flyers. These observations took place at baseline and 7–10 months after the start of the implementation, to assess which adjustments to the physical working environment were implemented and how they were implemented and to which extent visible communication, such as flyers or posters about activities was present.

#### Data analysis

A combination of both qualitative and quantitative data was used to determine the process indicators. Descriptive analyses were performed using the questionnaire data. Interviews with employers and employees were recorded and transcribed verbatim. The transcripts were coded by means of thematic coding. An initial codebook was drafted based on the applied frameworks, i.e. Wierenga, Nielsen & Randall, CFIR and COM-B, and new codes could emerge from the data (S3 Table). The first two transcripts were coded independently and discussed by two researchers. The remaining transcripts were coded by one researcher and checked by the other. Next, all coded transcripts were discussed until consensus between the two researchers was reached. If necessary, a third researcher was consulted. Information from the monitoring charts was used to determine the number of activities implemented within the different health behaviors on both the individual and organizational level. Information about adjustments to the physical working environment was derived from the checklists completed during the observations. To assess the dose received, the percentage of employees that participated in at least one individual-based activity and the percentage of employees that were exposed to an adjustment at the organizational level was determined. In case employees participated both in an individual-based activity and were exposed to an adjustment at the organizational level for at least two health behaviors, they were considered as compliant to the integrated WHPP and thus received the complete WHPP. MAXQDA software was used to analyze the qualitative data and analyses of quantitative data were performed using the Statistical Package of Social Sciences version 28.0 (SPSS Inc, Chicago, IL).

## Results

The questionnaire at six months follow-up was completed by 81 employees (90%). Mean age of the participants was 42.5 (11.6) years and 57.8% was female. Three organizations each delivered one monitoring chart. One organization had three local working groups and thus delivered three separate monitoring charts, leading to a total of six monitoring charts. A total of 11 employees across the 4 participating organizations were interviewed, of which three were part of the working group of their organization. Eight employers, all involved in working group of their organization were interviewed, details are depicted in Tables 2 and 3.

**Table 2:** Information about interviewees (employees).

Employees	Organization	Male/female
**1**	1	Female
**2**	1	Male
**3**	2	Male
**4**	2	Male
**5**	3	Male
**6**	3	Male
**7**	3	Female
**8**	4	Male
9^a^	1	Female
10^a^	2	Female
11^a^	2	Female

^a^ Employees who were part of the working group of their organization.

**Table 3:** Information about interviewees (employers).

Employers^a^	Organization	Male/female	HR/supervisor
**1**	1	Female	HR
**2**	1	Female	Supervisor
**3**	2	Female	HR
**4**	2	Female	HR
**5**	2	Female	Supervisor
**6**	3	Female	Supervisor
**7**	3	Female	Supervisor
**8**	4	Male	HR

^a^ Human Resources (HR) professionals and/or supervisors.

### Implemented activities

The implementation of activities on both the individual and organizational level for multiple health behaviors was a key element of the integrated WHPP. Nevertheless, none of the organizations met the criteria of the integrated WHPP six months after the start of the implementation. Organizations did implement activities within at least two health behaviors, but the activities were implemented not on both the individual and organizational level. Two organizations reached almost full compliance to the integrated WHPP at six months. They implemented activities at both the individual and organizational level for one health behavior, and an activity implemented on either the individual or organizational level for another health behavior (S4 Table). Another organization implemented one activity on the individual level, that encompassed multiple health behaviors. As none of the organizations managed to implement the integrated WHPP as intended at six months follow-up, the percentage of employees that received the integrated WHPP was zero. Eight to ten months after the start of the implementation, additional activities were implemented by two organizations. However, the criteria of the integrated WHPP were still not met (S4 Table). Activities at the organizational level mainly consisted of adaptations to the work environment, rather than policy adjustments. Based on the observations at the workplace, it appeared that all of the adaptations to the physical work environment, such as replacement of sodas, were implemented as reported. Possible explanations for not meeting the criteria of the integrated WHPP lie within the implementation process, which is described through the degree of implementation, perceptions of stakeholders, and contextual factors within four relevant themes i.e. composition and functioning of the working group, engagement and participation of employees, the role of management and policy and organizational preconditions.

### Composition and functioning of the working groups

One of the key elements of the integrated WHPP was the working group, consisting of HR, supervisors and employees, that selected and implemented activities from the catalogue. The working group could also create awareness about the activities and promote participation.

#### Degree of implementation

In each organization working groups were composed to implement the integrated WHPP. The interviewed employers indicated that contact persons in the organization sent out a call through various channels to recruit members for the working groups. They also approached employees and supervisors personally. Each working group had one or two group leaders, which were set by the members themselves. The division of roles within the working group, e.g. planning, execution, leadership, overall came naturally, such as on the basis of the role someone occupied within the organization. During meetings of working groups, ideas for health promotion activities were generated and discussed, achievements were evaluated and the implementation process was planned. All of the working groups used the catalogue to get inspired and to select activities. Overall, working groups collectively–during the working group meetings–selected activities from the catalogue within the health behaviors of interest, based on how easy they could be implemented. Working groups either used the implementation plan to check their progress or used it as a tool to initiate the process, but did not systematically follow every step. As a result, not all steps were conducted. For instance two out of four organizations identified health behaviors of interest by conducting a needs assessment among employees by disseminating an (online) survey. The other two organizations identified relevant health behaviors with the members of the working group. Mainly due to time constraints they did not conduct a needs assessment. However, the needs assessment was expected to be of added value, because of the insights it yielded regarding relevant health behaviors and suitable timing of activities according to both employees and employers. The working group informed employees about the activities through email (56%), internal websites (21%) and newsletters (17%) according to questionnaire data.

#### Perceptions of stakeholders (working group members)

Working group members were positive about the integrated WHPP. They recognized the working group as an essential and helpful component of the integrated WHPP:

*"Researcher: If you could mention one aspect of the program that has been most helpful,* *what would it be?*

*Employer*: *Those working groups*. *Absolutely*! *Despite what I just said*, *that it [number of implemented activities] is not enough*. *But you create ambassadors throughout the organization through such a working group*, *so to speak*. *Even if no concrete actions would have emerged*, *at least it initiates discussions*. *And I think that’s already a major success*. *So*, *despite the limited outcomes in activities*, *I truly believe it makes a meaningful contribution*.*"–Employer 8*

In some cases, enthusiasm about the integrated WHPP had to grow. Two working group members indicated that at the start they were skeptical about the integrated WHPP and expected that implementation of activities would entail a lot of work. However, during the implementation process and upon viewing the catalogue their view about the integrated WHPP changed in a positive way:

*“You see, initially, you think: oh, what do we have to do? But then, when you take a look at that intervention [catalogue], that menu you [researcher] provided, you realize that you’ve accomplished something fairly rapidly, I believe*. *It’s actually quite accessible. You should not complicate it too much.”–Employer 7*

Working group members indicated that the catalogue served as a source of inspiration and that it was a clear and comprehensive tool. Moreover, the categorization into themes was perceived to be convenient:

*“Well, I found the toolkit [catalogue] to be very comprehensive. I also appreciated that she [researcher] organized it into different categories, so you can see: what happens in each category? And the toolkit was more accessible than I had expected*. *Many of the interventions listed in it were actually quite easy to implement. Because we had a limited budget and little support, it made things [implementation] easier.”–Employer 6*

#### Contextual factors

Working group members mentioned in the interviews that they did not always had the opportunity to implement activities, primarily due to a lack of time. They implemented the activities in addition to their regular work.

*"Everyone recognizes its [vitality at work] importance, but daily priorities are simply being set*. *And that’s unfortunate. So is it a matter of time, or is it a matter of workload? Well, that’s the question. But because of that, we do fall behind, that’s for sure."–Employer 8*

Despite their limited time, working group members were enthusiastic and motivated to work with the integrated WHPP were hoping to dedicate more time to it. A perceived facilitator by working group members, was collaborating in a working group consisting of various representatives within the organization, i.e. employees, supervisors and HR-professionals:

“*I believe it’s great when within a group, you have things to dream about, to think through, to explore possibilities. But it’s also very valuable to have people who truly consider the reality, like what is feasible and what is not*. *I see that reflected in the working group as well.”–Employer 1*

A barrier according to employers was that composing the working groups within the organization was time consuming, amongst others as it was not always clear who was in charge and due to high work demands. As a result, several months in which activities could have been implemented had been lost. Moreover, (not) having working group members with specialized knowledge and in the right places, both physically (at a particular location, or a floor in a building) and in terms of position in the organization (e.g., a supervisor, head of department or office manager) could either hamper or support implementation of the integrated WHPP.

### Engagement and participation of employees

Employee engagement was essential in the implementation of the integrated WHPP. Hence, the implementation plan emphasized the involvement of employees in the working group and the necessity of assessing their needs regarding activities and timing which was expected to improve participation.

#### Degree of implementation

With regard to the awareness and participation of employees about the integrated WHPP, 72% of the employees indicated that they were aware of the integrated WHPP that was implemented in the context of the study. However, only 14% and 16% of employees used the environmental adjustments or participated in activities respectively. During interviews, employees were unable to recall implemented activities. However, when implemented activities were listed by the researcher, employees recognized and, in some cases, had participated in them.

#### Perceptions of stakeholders (employees)

Employees who were a member of the working group mentioned that they enjoyed being a part of it, as they felt involved. They also indicated that they were more inclined to set a positive example. However, one employee who was not part of the working group noticed how involved the working group was, but mentioned that this did not extend beyond the working group:

*"Interviewer: Do you feel that employees were involved in the process* *of selecting and implementing activities?*

*Employee*: *No*. *No*, *I haven’t heard much about that*. *I believe that’s something that stayed within the working group mostly […]*. *Well*, *that remained quite internal*, *I think*.*"–Employee 4*

**Fig 2:**
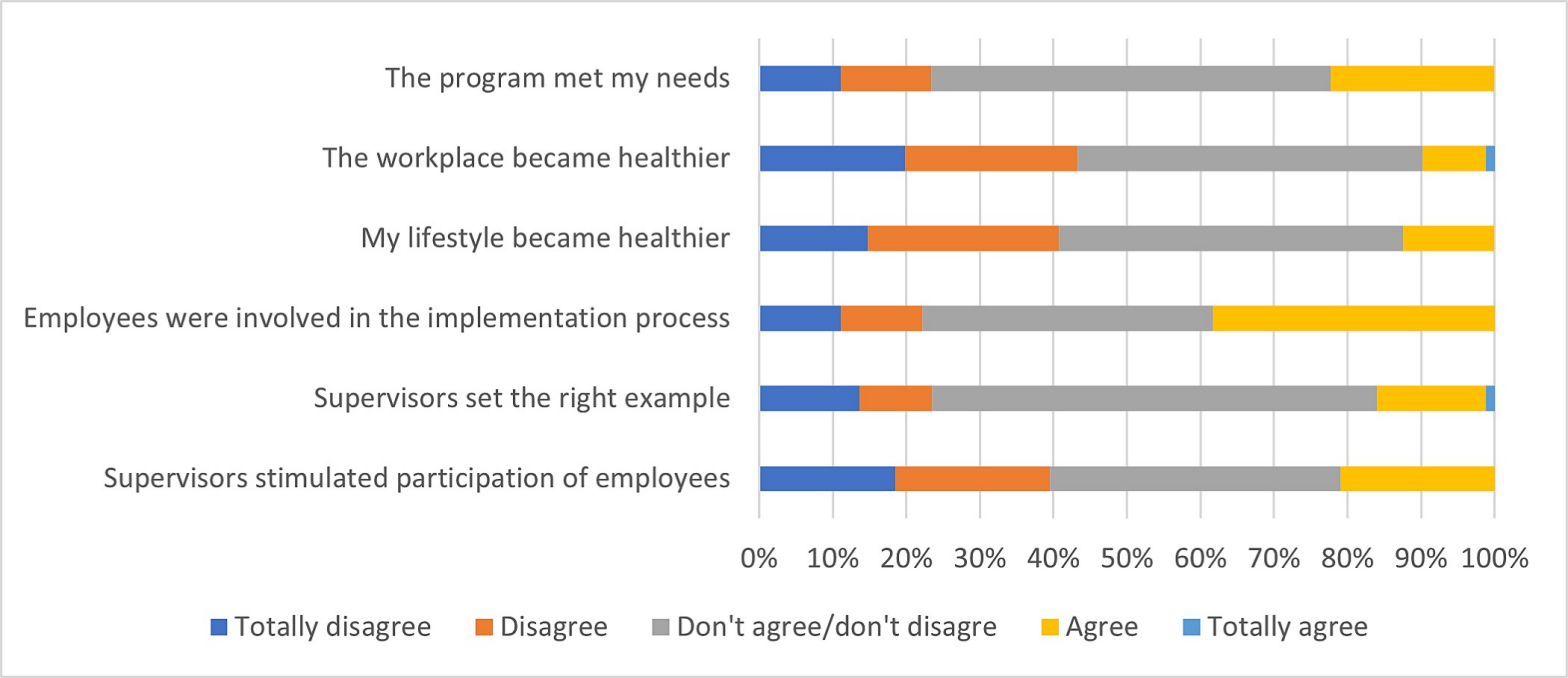
Questionnaire data regarding opinion and appraisals of employees about the integrated WHPP.

The questionnaire, completed by employees, provided information about the opinions and appraisals of the employees about the integrated WHPP. In the questionnaire, employees rated the integrated WHPP with a 6.4 (2.2) on a scale of 1–10 on average. Results from the questionnaire also imply that almost a quarter of the employees (23%) did (totally) disagree with the statement that the integrated WHPP met their needs. Less than half of the employees (44%) indicated that the workplace did not become healthier, and a comparable percentage (41%) reported that they did not adopt a more healthy lifestyle. 22% of the employees did not feel involved in the implementation process. Fig 2 shows results on opinions and appraisals of employees about the integrated WHPP.

In the interviews, employees indicated that only a small number of activities was implemented, which they found unfortunate. However, they were satisfied with the non-committal nature of the integrated WHPP, as this allowed them to choose whether they wanted to participate in implemented activities or not. When participation is imposed or obligatory, it can actually hinder participation:

*“So I don’t necessarily want something imposed on me*. *I still want to be able to make a choice. And as long as I can make that choice, nine times out of ten, I’ll try to make the right one. But I’ve had the experience myself: if something is going to be imposed on me, then I become stubborn”–Employee 1*

#### Contextual factors

**Fig 3:**
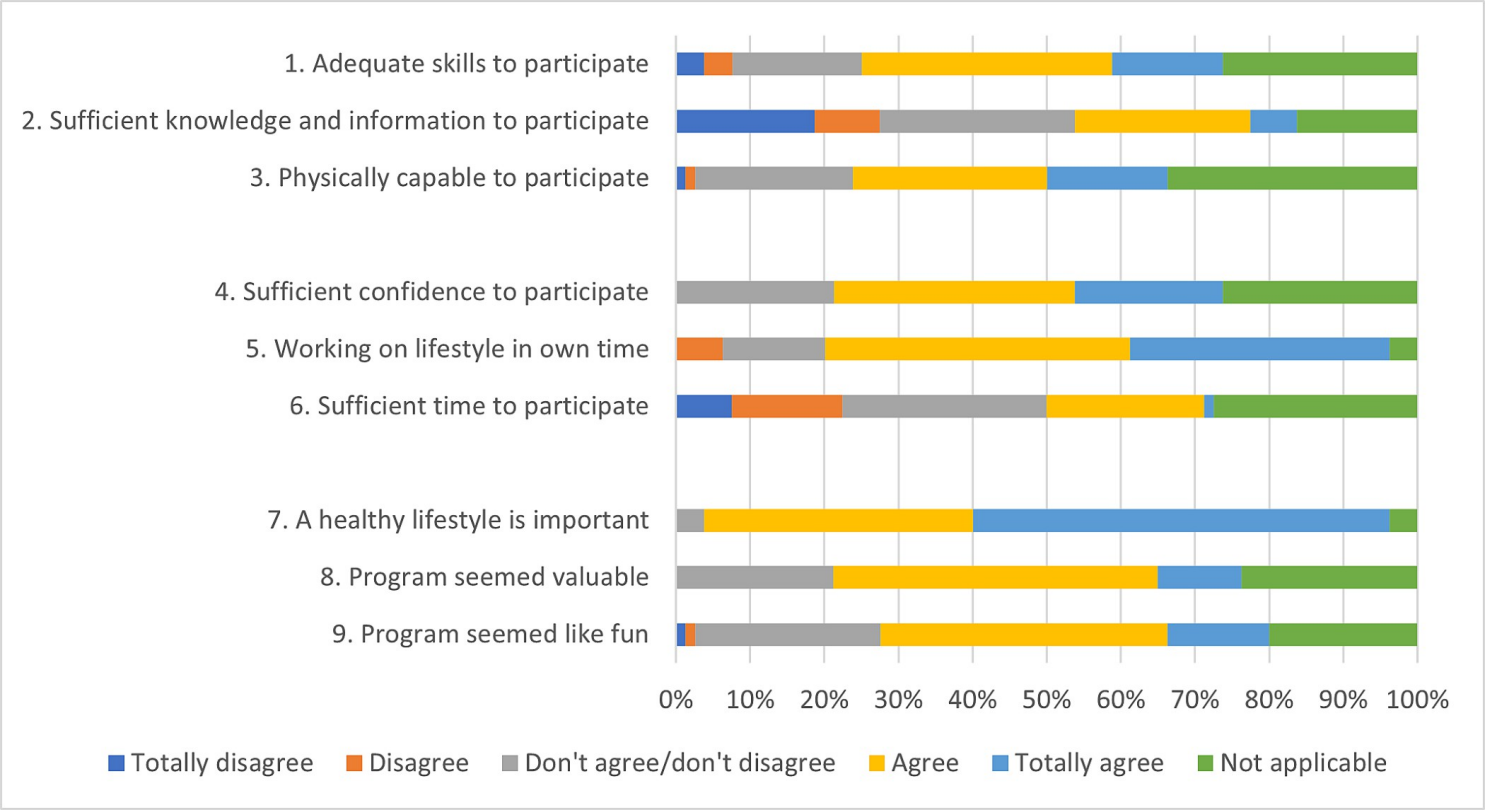
Employees’ capability (1–3), opportunity (4–6) and motivation (7–9) to participate in activities, based on the COM-B model.

Contextual factors such as opportunity and timing of activities affected the participation of employees in the implemented activities. Questionnaire data indicated that 30% of the employees had sufficient knowledge and information to participate in activities. Almost a quarter (23%) of the employees did not have sufficient time to participate in implemented activities and a majority of the participants indicated that they already have a healthy lifestyle (76%). Almost all employees (92%) agreed with the statement that a healthy lifestyle is important, which was therefore perceived as an important motivation to work on a healthy lifestyle (Fig 3).

The scheduling of activities was perceived as a barrier for participation. Employees indicated that they did not participate in activities that did not take place adjacent to working hours.

### The role of management and policy

Based on information from both questionnaires and interviews, the role of management i.e. higher management and supervisors, and organizational policies appeared to be important in the implementation of the integrated WHPP.

#### Perceptions of stakeholders (employees)

Regarding the role of supervisors, it became apparent that an adequate balance has to be found in stimulating participation in WHPPs. That is, as soon as employees feel that something is imposed or decided by their supervisors, or that there is some form of peer pressure from colleagues, they are not willing to participate anymore:

*“But as soon as there’s actually some kind of expectation expressed by a supervisor, I think that’s very quickly interpreted as if your supervisors, from his position, also expects you to do this and that you’re not doing your job well if you don’t participate*. *And yes, that’s the line that I think you shouldn’t cross.”–Employee 6*

In the questionnaire, 40% and 24% of the employees (totally) disagreed with the statement that supervisors stimulated participation of employees in activities implemented as part of the integrated WHPP and that supervisors set the right example, respectively (Fig 3).

#### Contextual factors

A barrier that affected the implementation of the integrated WHPP was the absence of organizational policies regarding vitality, e.g. including vitality activities in organizational long-term plans, encouraging cycling to work by providing incentives or compensations, ensuring a healthy caterer for the company restaurant and organizational events. For this reason, activities were perceived by employees as separate initiatives without consistency, which was perceived as a barrier for participation:

*"I expected that there would be more structured events at specific times, including after working hours, to make it more dynamic. In my opinion, it’s not really a cohesive program where you can engage seamlessly. They are more like isolated activities*. *I expected more coherence"–Employee 6*

Nevertheless, some employers thought of the implementation of the integrated WHPP as a proactive signal to the organization, emphasizing the value of WHP through concrete actions while others observed increased awareness and recognition of WHP within the organization as a result of the integrated WHPP. Overall, working group members felt support from higher management, e.g. the board of directors, which was identified as a facilitator for implementation. Yet, according to working group members, a more active role of higher management could induce more effect, e.g. by setting a good example.

### Organizational preconditions

Lastly various organizational preconditions appeared required for successful implementation of and participation in the integrated WHPP, not all of them were present in the participating organizations.

#### Perceptions of stakeholders (employers and employees)

The organizational culture and employees’ willingness to engage are examples of organizational preconditions. Employees in organizations were generally positive about the organization offering WHP, and are open to participating, given the availability of time, which was not always the case. Furthermore, overall lifestyle, comprising a combination of different health behaviors, was seen as a personal choice and responsibility. Although, employers do have a responsibility to facilitate a healthy lifestyle for employees by providing opportunities, e.g. options to exercise at work or get a healthy lunch, and ensuring a healthy environment, e.g. with fruit, sit-stand desks or lunchtime walks. However, imposing these measures would hinder participation.

*“I think so, although it may quickly be seen as intrusive*. *But I do think that an employer can provide tools, offer opportunities like: we offer you the opportunity, for example, to talk to a sports coach or a dietitian. I believe that an employer can definitely play an important role in that.”–Employee 8*

The bureaucracy within the organizations challenged the implementation of the integrated WHPP according to multiple employers. For example, when placing stickers to promote taking the stairs, meeting numerous requirements and navigating through various layers of the organization were necessary before actual implementation could occur. During the implementation process one employer recognized the importance of embedding activities and plans in the annual or long-term plans.

#### Contextual factors

For successful implementation local needs within the organization or working location should be considered. Employers indicated that the multifaceted nature of the integrated approach enabled them to tailor activities to preferences and needs of employees, which facilitated implementation. Almost all employers reported the research context as a barrier for implementation, as the presence of a control condition, restricted the number of communication channels that could be used. Additionally, some activities, such as adjustments to the physical work environment or implementation of policies, were difficult to implement in the intervention condition only. Hence, working groups strived to select activities that could be implemented in the intervention condition only and opted for communication channels exclusive to the working locations in the intervention condition. Moreover, employers indicated that it was hard to accept that they could not offer activities to all employees, since sick leave rates also had to be reduced in the control condition. On the other hand, organizations have taken initial steps in implementing WHP, due to the study. Despite the challenges with the implementation, all of the organizations had the intention to continue with the implementation of the integrated WHPP. In doing so, they indicated that policies regarding vitality, greater awareness and familiarity, time and budget are necessary.

## Discussion

The aim of this study was to evaluate the implementation process of the integrated WHPP by assessing the degree of implementation, perceptions of employees and employers and contextual factors affecting implementation. Based on the findings, it can be established that the key elements of the integrated WHPP were not entirely manifested in practice, several factors within four themes relevant to the implementation process i.e. the composition and functioning of the working group, engagement and participation of employees, the role of management and policies and organizational preconditions, contributed to this. Although organizations were able to establish a working group consisting of HR professionals, supervisors and employees, this process entailed a considerable amount of time, which substantially delayed the implementation of activities. Additionally, working group members indicated that they experienced a lack of time to actually select and implement the activities. A positive factor was that working group members were motivated and satisfied with the integrated WHPP. They reported the multifaceted nature of the integrated WHPP as a facilitator and the absence of policies regarding vitality as a barrier for implementation. Overall, the process of composing a working group and selecting and implementing four activities according to the criteria of the integrated WHPP within six months, was not feasible for the organizations.

From our findings, it appeared that both working group members and employees did not always had the opportunity to implement or participate in activities respectively, which was mainly due to a lack of time and/or other priorities. As this might be related to the organizational culture and the absence of policies concerning vitality, a shift to a culture where (working on) a healthy lifestyle is the standard and not the exception, is essential [25–28]. In our study, the absence of organizational policies concerning vitality also made it challenging to implement activities with regularity and coherence, which is another factor that underlines the need for such policies. These policies might involve including vitality activities in organizational long-term plans, encouraging cycling to work by providing incentives or compensations, ensuring a healthy caterer for the company restaurant and organizational events and can contribute to a supportive organizational culture regarding WHP [29]. In the current integrated WHPP activities at the organizational level could either comprise adjustments to the social or physical working environment or adjustments to policies, making the adjustments of policy optional. To enhance the implementation of policies regarding health and vitality, it might be beneficial to incorporate a mandatory component concerning policy adaptation in the integrated WHPP, eliminating the optional aspect.

The implementation of policies requires actions of higher management, which is why commitment and support of higher management towards WHP is of importance [30]. In our study, higher management was committed and did approve the implementation of activities in the organization. However, employers indicated that a more active role of higher management, e.g. active involvement in shaping the WHP offer, embed vitality in policies and the mission of the organization and actively promoting the importance of vitality within the organization, in the implementation could potentially enhance the success of the implementation. Multiple other studies underline the importance of active support from higher management, on both the level of implementation and participation of employees in activities [6, 30–36]. A WHPP in Andalusia, Spain, also based on the Lombardy Workplace Health Promotion Network (LWHPN), identified commitment of higher management as a strength for the implementation as they allowed employees to dedicate working time to participate in activities [37]. In future studies, it is therefore recommended to promote active involvement and continuous commitment of higher management in the implementation of a WHPP. An accreditation process, included in both the LWHPN and Andalusian WHPP might also have accounted for a boost in motivation of higher management to implement the WHPP. Therefore it might be valuable to include this in future updates of the integrated WHPP under study [37].

Also factors positively affecting the implementation were observed in this study. Even though it took a significant amount of time to get the working group up and running, members of the working group valued working together on vitality in a multidisciplinary team. Employees in the working group felt involved, which is known to positively affect their engagement in WHP [30, 35, 38]. Moreover, members of the working group were very motivated to work on this project and individual characteristics such as motivation and commitment are known to attribute to an individual’s suitability of implementing and maintaining WHP activities [32]. However, only a limited number of employees can be directly engaged in a working group. Moreover, results of our study indicated that the working groups were not that visible for other employees and that their enthusiasm did not always transfer to the other employees. Hence, additional efforts may be necessary to adequately involve other employees. This might involve utilizing regular meeting times to identify employee needs in a more personalized manner than through a questionnaire, ongoing communication, utilizing various communication channels, about what is done with these identified needs and increasing the visibility of the working group so that the threshold for employees outside the working group to submit ideas is lower. For future implementation of WHP the formation of a working group with motivated members is encouraged as it might improve the reach, participation and support of the WHPP throughout the organization, on the condition that working group members clearly present themselves as ambassadors [17, 18, 30, 39].

Employees rated the integrated WHPP with a 6.4 out of 10. A possible explanation for the fact that the integrated WHPP was not graded higher is that initial steps towards WHP were taken and the program therefore was not always visible to the employees. The low participation rate also implies a lack of awareness of employees about the specific activities implemented in the context of the study. Moreover, the low number of implemented activities and the low percentage (22%) of employees that felt their needs were met, might account for the relatively low rating of the integrated WHPP by employees. To align more closely with the needs of employees, a more diverse set of activities could be implemented based on a proper needs assessment. Even though a needs assessment was recommended in the implementation plan, only two organizations actually conducted a needs assessment [17, 18]. Another previously identified facilitator that potentially lacked attention during the implementation of the integrated WHPP, was support from supervisors [17, 18]. This might be partially attributable to the adequate balance that has to be found for supervisors in stimulating participation in WHP without imposing it. Informing supervisors about implemented activities and raising their awareness with regard to the role-model position they have is an important task of the working group which requires further attention in future implementation of WHPPs. This might be achieved by providing supervisors with a training about their role regarding vitality at work, how they can stimulate employees to participate in activities, without imposing it and to enhance their ability to serve as a role model [40].

### Strengths and limitations

A strength of this study was the mixed methods study design. The combination of data from interviews, questionnaires, monitoring charts and observations provided us insight in the implementation process of the integrated WHPP. Moreover, it was observed that data saturation was achieved in the interviews, indicating that sufficient information was collected. Another strength is that the integrated WHPP was implemented in four varying occupational settings, enriching the data as different factors within each implementation component were observed. As mentioned in the introduction, Murta et al. (2007) reported a high number of process evaluations being incomplete, lacked a theoretical framework and were not planned prior to implementation [12]. As the current process evaluation has been reported following two frameworks, was planned in advance and is complete, by reporting on the degree of implementation, perceptions of stakeholders and contextual factors, this can be seen as a strength. A limitation is that the implementation of the integrated WHPP is potentially affected by the C-RCT conducted to analyze the effect of the integrated WHPP on overall lifestyle, which is also reported in other studies [41, 42]. For instance, employers indicated that implementation was difficult because of the control condition, especially on the organizational level. Additionally, they experienced difficulties in communication as many channels were used organization-wide and are linked together. Moreover, employers experienced time pressure from the study, due to scheduled measurement moments. Resulting in an unnatural process and a focus on the practical part of the implementation, rather than tailoring activities to the needs of employees. For future studies, it may be worthwhile to consider other study designs than an RCT, such as a stepped wedge design, where all participants receive the intervention [41, 42]. Another limitation is potential bias in the questionnaire, as employees might have confused the integrated WHPP (intervention) with the research they participated in (e.g. answering questions about communication from the research perspective instead of the intervention perspective or vice versa). Moreover, it is possible that there was selection bias as participants in this study might be predominantly employees with a positive attitude towards WHPPs. Participating organizations were motivated and willing to implement the integrated WHPP and employed predominantly highly educated employees, potentially leading to selection bias and limited generalizability, especially given the limited number of participating organizations.

## Conclusion

This process evaluation indicates how challenging and complex successful implementation of WHPPs in organizations is. Important lessons learned for implementation of (integrated) WHPPs include the importance of organizational policies concerning vitality, and the necessity for implementers to have sufficient time and thus the opportunity to prioritize the implementation of activities. Despite this challenged implementation of the integrated WHPP in practice, the program was well received by the working groups and they all had the intention to continue with the implementation. Achievements mentioned by the working groups, such as stressing the importance of WHP through concrete actions and facilitating conversations about workplace vitality, are important first steps for a successful implementation of WHP. However, based on insights from this process evaluation, an organizational culture where attention to vitality is part of the organization’s identity is needed to successfully implement WHP and to make a greater impact on the targeted health behaviors of employees.

## Supporting information

S1 Table Characteristics of the participating organizations. (PDF)

S2 Table Template of a monitoring chart. (PDF)

S3 Table Codebook with both initial and newly emerged codes. (PDF)

S4 Table Overview of activities implemented 6–10 months after the start of the implementation. (PDF)
