# Supplementary material for: The implementation of an integrated workplace health promotion program in Dutch organizations ‐ A mixed methods process evaluation
Source: PLoS One. 2024 Nov 1;19(11):e0308856. doi: 10.1371/journal.pone.0308856 (PMC11530008; doi:10.1371/journal.pone.0308856)
Supplement: S1 Table — (PDF) [file pone.0308856.s001.pdf]

S1 Table. Characteristics of the participating organizations

Organization	Occupational sector	Number of employees	Locations
1	Educational organization	319[a]	2
2	Assurance, tax and consulting organization	639	7
3	Educational organization	197	4
4	Retail organization	256[a]	27[b]

[a] Number of employees in participating departments within the organization. [b] Divided over four regional clusters.
