# Supplementary material for: The implementation of an integrated workplace health promotion program in Dutch organizations ‐ A mixed methods process evaluation
Source: PLoS One. 2024 Nov 1;19(11):e0308856. doi: 10.1371/journal.pone.0308856 (PMC11530008; doi:10.1371/journal.pone.0308856)
Supplement: S2 Table — (PDF) [file pone.0308856.s002.pdf]

S2 Table. Template of a monitoring chart

**Implemented activity**	
Name activity	
Short description activity	
**Lifestyle themes**	
Select the lifestyle theme(s) targeted by the activity	
**Levels**	
Select the level (individual or organizational) on which the activity is implemented	
**Time**	
How long did the implementation process take	
Date: start of implementation	*Date: activity implemented*
How many hours per day/week/month were spent on the implementation	
Hours	*Per day/week/month*

**Communication to employees**	
Describe how employees were informed about the activity	
**Sessions**	
If applicable: how many sessions were included in the activity	
Number of sessions	
How many employees participated in each session	
Session	*Number of participants*
