# Supplementary material for: The implementation of an integrated workplace health promotion program in Dutch organizations ‐ A mixed methods process evaluation
Source: PLoS One. 2024 Nov 1;19(11):e0308856. doi: 10.1371/journal.pone.0308856 (PMC11530008; doi:10.1371/journal.pone.0308856)
Supplement: S3 Table — (PDF) [file pone.0308856.s003.pdf]

S3 Table. Codebook with both initial and newly emerged codes

Level 1	Level 2	Level 3	Level 4	Level 5	Explanation
**General information**	**Years of working for the organization**				Introduction
	**Function**				Introduction
	**Organization**				Introduction
		**1**			Introduction
		**2**			Introduction
		**3**			Introduction
		**4**			Introduction
	**Employee**				
	**Employee (working group)**				Employee who is part of the working group
	**Employer**				HR or supervisor (part of the working group)
**Degree of implementation**	**Implementation strategy**				Roles and behaviors of key stakeholders (employer perspective)
		**Selection of activities***			The process of selecting activities from the catalogue by the working group
		**Formation of the working group***			The process of forming the working group
		**Familiarity with the working group***			To what extent were employees familiar with the working group, and what did the working group do

Level 1	Level 2	Level 3	Level 4	Level 5	Explanation
					to increase familiarity
		Task distribution within the working group*			How were the tasks distributed among the working group members
	Fidelity				Compliance (implemented as intended)
		Integrated approach			Met the criteria of the integrated WHPP
		Materials of the integrated WHPP (Implementation plan/catalogue)			Were the steps of the implementation plan systematically followed, was the catalogue used to select activities
	Dose delivered				Number of activities that is implemented by The employer (working group)
	Dose received				The extent to which employees participated in activities
	Recruitment				Sources and procedures used to stimulate participation
		Materials			Which communication materials were used (poster, mail, presentation)

Level 1	Level 2	Level 3	Level 4	Level 5	Explanation
		Individuals			Which individuals were deployed (supervisors, ambassadors)
			Rationale and motivation*		Reason why (and how) specific individuals were deployed
	Reach				Proportion of employees who were aware of implemented activities
**Perceptions of employees and employers**	Satisfaction	Employees			Satisfaction about implemented activities
		Employers			Satisfaction about implementation process of activities.
			Satisfaction about own achievements and performance*		To what extent were employers satisfied with their own accomplishment and why
			Satisfaction about integrated WHPP*		Were the employers satisfied about the integrated WHPP (approach and materials)
	Participants mental models	Employees			Perceptions and appraisals about the program (employee perspective)<br>- Overall attitude towards the concept

Level 1	Level 2	Level 3	Level 4	Level 5	Explanation
			Added value to be part of the working group*		Did employees considered being part of the working group to be of added value
			Getting something imposed vs personal choice*		To what extent did employees perceive that activities were imposed, and why. And what did they prefer in this regard
			Reactions within working environment*		How did individuals in their working environment respond to activities and what did this do for their own motivation or attitude
				Supervisors*	Reactions of supervisors regarding the activities
				Colleagues*	Reactions of colleagues regarding the activities
			Experiences*		What did their experiences with (prior) activities do with their motivation or attitude
			Needs and preferences*		Were needs and preference met, and what did this do to their

Level 1	Level 2	Level 3	Level 4	Level 5	Explanation
					motivation and attitude
			**Attitude/belief ***		What was their attitude towards or belief in activities (prior to the study and during the study)
			**Predefined goals***		Did predefined goals (such as the will to improve lifestyle) affect their motivation, intention or attitude
			**Expectations***		What were their expectations and how did these affect their motivation, intention or attitude
		**Employers**			Perceptions and appraisals about the program (employer perspective)
			**New insights/awareness***		New insights or awareness about WHPP, as a result of the integrated WHPP (and its materials)
			**Responsibility***		Did employers feel responsible, and how did this affect their will to implement activities

Level 1	Level 2	Level 3	Level 4	Level 5	Explanation
			**Reactions within working environment***		How did employees respond to activities, and how did this affect their motivation or will to implement
			**Belief***		Did they belief in the integrated WHPP and how did this affect their will to implement activities
				**Self-efficacy***	Did they belief they were able to implement activities
			**Experiences***		What were their experiences regarding implementation and how did it affect their attitude/motivation/will to implement
			**Expectations***		What were their expectations and how did they affect their attitude/motivation/will to implement
			**Predefined goals***		What were their goals regarding the implementation of activities, and how did that affect their attitude/motivati

Level 1	Level 2	Level 3	Level 4	Level 5	Explanation
					on/will to implement
**Contextual factors**	**Context**				Barriers and facilitators for participation and implementation
		**Innovation domain**	**Costs**		The innovation purchase and operating costs are affordable. (not including mentions about location and time)
			**Design**		The innovation is well designed and packaged, including how it is assembled, bundled, and presented
				**Visibility of the activity***	Were activities visible enough for employees
				**Timing of the activity***	Was the timing of activities convenient
				**Structure of the activity***	Coherence of implemented activities
				**Improvements of materials of the integrated WHPP***	Suggestions for improvements of materials of the integrated WHPP
			**Complexity**		The innovation is complicated, which may be reflected by its scope and/or the nature and number of connections and steps
			**Trialability**		The innovation can be tested or piloted on a small scale and undone
			**Adaptability**		The innovation can be modified, tailored, or

Level 1	Level 2	Level 3	Level 4	Level 5	Explanation
					refined to fit local context or needs
			Relative advantage of the intervention		The innovation is better than other available innovations or current practice
			Intervention is evidence based		The innovation has robust evidence supporting its effectiveness
			Intervention source		The group that developed and/or visibly sponsored use of the innovation is reputable, credible, and/or trustable
		Outer setting domain	External pressure	Performance-measurement-pressure	Quality or benchmarking metrics or established service goals drive implementation and/or delivery of the innovation
				Market pressure	Competing with and/or imitating peer entities drives implementation and/or delivery of the innovation
				Societal pressure	Mass media campaigns, advocacy groups, or social movements or protests drive implementation and/or delivery of the innovation
			Financing		Funding from external entities (e.g., grants, reimbursement) is available to implement and/or deliver the innovation

Level 1	Level 2	Level 3	Level 4	Level 5	Explanation
			Policies and laws		Legislation, regulations, professional group guidelines and recommendations, or accreditation standards support implementation and/or delivery of the innovation
			Partnerships and connections		The Inner Setting is networked with external entities, including referral networks, academic affiliations, and professional organization networks
				The study*	Interference with the study design (the cluster randomized controlled trial to assess effectiveness of the integrated WHPP)
			Local conditions		Economic, environmental, political, and/or technological conditions enable the Outer Setting to support implementation and/or delivery of the innovation
			Local attitudes		Sociocultural values (e.g., shared responsibility in helping recipients) and beliefs (e.g., convictions about the worthiness of recipients) encourage the Outer Setting to support implementation

Level 1	Level 2	Level 3	Level 4	Level 5	Explanation
					and/or delivery of the innovation
			Critical incidents		Large-scale and/or unanticipated events disrupt implementation and/or delivery of the innovation
		Inner setting domain	Available resources	Access to knowledge and information	Guidance and/or training is accessible to implement and deliver the innovation
				Materials and equipment	Supplies are available to implement and deliver the innovation
				Space	Physical space is available to implement and deliver the innovation
				Funding	Funding is available to implement and deliver the innovation
				Time*	Time could be allocated to the implementation of activities
			Mission alignment		Implementing and delivering the innovation is in line with the overarching commitment, purpose, or goals in the Inner Setting
			Incentive systems		Tangible and/or intangible incentives and rewards and/or disincentives and punishments support implementation and delivery of the innovation.

Level 1	Level 2	Level 3	Level 4	Level 5	Explanation
			Relative priority		Implementing and delivering the innovation is important compared to other initiatives
			Compatibility		The innovation fits with workflows, systems, and processes
			Tension for change		The current situation is intolerable and needs to change
			Structural characteristics	Work infrastructure	Organization of tasks and responsibilities within and between individuals and teams, and general staffing levels, support functional performance of the Inner Setting
				Information technology infrastructure	Technological systems for tele-communication, electronic documentation, and data storage, management, reporting, and analysis support functional performance of the Inner Setting
				Physical infrastructure	Layout and configuration of space and other tangible material features support functional performance of the Inner Setting
				Current WHP offer*	What was the current offer of WHP activities
				Organizational policies*	Policies regarding vitality within the organization

Level 1	Level 2	Level 3	Level 4	Level 5	Explanation
			Culture	Learning centeredness	There are shared values, beliefs, and norms around psychological safety, continual improvement, and using data to inform practice
				Deliverer centeredness	There are shared values, beliefs, and norms around caring, supporting, and addressing the needs and welfare of deliverers
				Recipient centeredness	There are shared values, beliefs, and norms around caring, supporting, and addressing the needs and welfare of recipients
				Human equality centeredness	There are shared values, beliefs, and norms about the inherent equal worth and value of all human beings
				Support from colleagues*	There was support to participate in or implement activities from colleagues
				Support from supervisors*	There was support to participate in or implement activities from supervisors
				Support from higher management*	There was support to participate in or implement activities from higher management

Level 1	Level 2	Level 3	Level 4	Level 5	Explanation
			Communications		There are high quality formal and informal information sharing practices within and across Inner Setting boundaries (e.g., structural, professional)
			Relational connections		There are high quality formal and informal relationships, networks, and teams within and across Inner Setting boundaries (e.g., structural, professional)
		Individuals Domain	Innovation recipients	Motivation	The individual(s) is committed to fulfilling Role. (Includes motivation to participate in a particular activity)
				Opportunity	The individual(s) has availability, scope, and power to fulfill Role
				Capability	The individual(s) has interpersonal competence, knowledge, and skills to fulfill Role
				Need	The individual(s) has deficits related to survival, well-being, or personal fulfillment, which will be addressed by implementation and/or delivery of the innovation
			Innovation deliverers	Motivation	Individuals who are directly or indirectly delivering the innovation

Level 1	Level 2	Level 3	Level 4	Level 5	Explanation
				Opportunity	
				Capability	
				Need	
			Other implementation support	Motivation	Individuals who support the Implementation Leads and/or Implementation Team Members to implement the innovation
				Opportunity	
				Capability	
				Need	
			Implementation Team members	Motivation	Individuals who collaborate with and support the Implementation Leads to implement the innovation, ideally including Innovation Deliverers and Recipients
				Opportunity	
				Capability	
				Need	
			Implementation leads	Motivation	Individuals who lead efforts to implement the innovation
				Opportunity	
				Capability	
				Need	
			Implementation facilitators	Motivation	Individuals with subject matter expertise who assist, coach, or support implementation

Level 1	Level 2	Level 3	Level 4	Level 5	Explanation
				Opportunity	
				Capability	
				Need	
			Opinion Leaders	Motivation	Individuals with informal influence on the attitudes and behaviors of others
				Opportunity	
				Capability	
				Need	
			Mid-level leaders	Motivation	Individuals with a moderate level of authority, including leaders supervised by a high-level leader and who supervise others
				Opportunity	
				Capability	
				Need	
			High-level leaders		Individuals with a high level of authority, including key decision-makers, executive leaders, or directors
		Implementation process domain	Adapting		Modify the innovation and/or the Inner Setting for optimal fit and integration into work processes
			Reflecting and evaluation	Innovation	Collect and discuss quantitative and qualitative information about the success of the innovation

Level 1	Level 2	Level 3	Level 4	Level 5	Explanation
				**Implementation**	Collect and discuss quantitative and qualitive information about the success of implementation
				**Feedback from recipients***	The feedback of employees regarding the implemented activities
			**Doing**		Implement in small steps, tests, or cycles of change to trial and cumulatively optimize delivery of the innovation
			**Engaging**	**Innovation recipients**	Attract and encourage recipients to serve on the implementation team and/or participate in the innovation
				**Innovation deliverers (Project leader*)**	Attract and encourage deliverers to serve on the implementation team and/or to deliver the innovation. *project leader, someone working on WHP specifically
				**External parties***	Attract external parties to assist with the implementation of activities
				**Higher management***	Attract and involve higher management in the implementation of activities and WHP

Level 1	Level 2	Level 3	Level 4	Level 5	Explanation
			**Tailoring strategies**		Choose and operationalize implementation strategies to address barriers, leverage facilitators, and fit context
			**Planning**		Identify roles and responsibilities, outline specific steps and milestones, and define goals and measures for implementation success in advance
			**Assessing context**		Collect information to identify and appraise barriers and facilitators to implementation and delivery of the innovation
				**Inventory***	What is already available regarding WHP, and what is lacking
			**Assessing needs**	**Innovation recipients (methods*)**	Collect information about the priorities, preferences, and needs of recipients to guide implementation and delivery of the innovation. *methods, how was a needs assessment conducted
				**Innovation deliverers**	Collect information about the priorities, preferences, and needs of deliverers to guide

Level 1	Level 2	Level 3	Level 4	Level 5	Explanation
					implementation and delivery of the innovation
			**Teaming**		Join together, intentionally coordinating and collaborating on interdependent tasks, to implement the innovation
				**Persistent effort***	
				**Collaboration***	Working together in a multidisciplinary team
				**Members in the right places***	Are members in the right place, both physical (in a particular area within the building, or regarding their role within the organization)
				**Different directions***	Different opinions and preferences
				**Task distribution***	Importance of a proper distribution of task
				**Expertise***	Importance of including individuals with the necessary knowledge
				**Time consuming***	Time necessary to get a working group up and running
	**Maintenance**	**Organization**			What is necessary for continuation of the integrated WHPP within the organization
		**Intervention**			Which adaptions to the integrated WHPP are necessary in order to maintain

Level 1	Level 2	Level 3	Level 4	Level 5	Explanation
					the WHPP within the organization

*Codes that emerged from the data
