# Supplementary material for: The implementation of an integrated workplace health promotion program in Dutch organizations ‐ A mixed methods process evaluation
Source: PLoS One. 2024 Nov 1;19(11):e0308856. doi: 10.1371/journal.pone.0308856 (PMC11530008; doi:10.1371/journal.pone.0308856)
Supplement: S4 Table — (PDF) [file pone.0308856.s004.pdf]

**S4 Table. Overview of activities implemented 6-10 months after the start of the implementation**

| | | Organization 1 | Organization 2 | | | Organization 3 | Organization 4 |
|---|---|---|---|---|---|---|---|
| Location | | | 1 | 2 | 3 | | |
| **Physical activity** | *Individual level* | Infographic informing employees about the WHP offer regarding physical activity[a] | Microsoft Teams channel to schedule exercise activities with colleagues[a] | Microsoft Teams channel to schedule exercise activities with colleagues[a] | Microsoft Teams channel to schedule exercise activities with colleagues[a] | Exercise workshops organized by ambassadors[a] | |
| | | Motivating quotes at digital screens and posters[b] | | Encourage participation in a city run[b] | | Exercise activity after personnel day[a] | |
| | *Organizational level* | Nudges to increase use of | | | | Active personnel days[a] | More options (i.e. different sports) in |

| | | | | | | | |
|---|---|---|---|---|---|---|---|
| | | the stairs instead of the elevator (stairs)[b] | | | | Policy to include exercise activities, workshops and active personnel days in annual agenda plans[b] | how to spend the vitality budget[a] |
| **Nutrition** | *Individual level* | Infographic informing employees about the WHP offer regarding nutrition[a] | A tasting of healthy syrups (to replace sodas)[a] | A tasting of healthy syrups (to replace sodas)[a] | A tasting of healthy syrups (to replace sodas)[a] | | Information about the importance of healthy nutrition, accompanied by vitamin C tablets[b] |
| | | Motivating quotes at digital screens and posters[b] | | | | | |

| | | | | | | | |
|---|---|---|---|---|---|---|---|
| | *Organizational level* | | Healthy syrups and a water tap with cold and sparkling water to replace sodas[a] | Healthy syrups and a water tap with cold and sparkling water to replace sodas[a] | Healthy syrups and a water tap with cold and sparkling water to replace sodas[a] | Active personnel days[a] | |
| | | | Healthy options during lunch[a] | An expansion of fruit offered at the workplace, both in diversity and quantity[b] | | Policy to include active personnel days in annual agenda plans[b] | |
| | | | Healthy snacks during meetings and gatherings[a] | | | | |
| **Mental balance** | *Individual level* | Infographic informing employees about the WHP offer | Promote employees to support colleagues participating in a city run[a] | | | | Information on regulations and opportunities around taking leave[a] |

| | | | | | | | |
|---|---|---|---|---|---|---|---|
| | | regarding mental balance[a] | | | | | |
| | | Motivating quotes at digital screens and posters[b] | | | | | Availability of a budget coach[a] |
| | *Organizational level* | | | 'Shuffle Tuesday' everyone sits at a different desk to meet other colleagues[b] | | | Informing and stimulating supervisors by management to have an open dialogue on work-life balance with employees (including taking days off)[a] |
| **Smoking** | *Individual level* | | | | | | |

| | | | | | | | |
|---|---|---|---|---|---|---|---|
| | *Organizational level* | | | | | | |
| **Alcohol** | *Individual level* | Infographic informing employees about the WHP offer regarding alcohol consumption[a] | | | | | |
| | | Vitality quotes at digital screens and posters[b] | | | | | |
| | *Organizational level* | | | | | | |
| **Sleep** | *Individual level* | Motivating quotes at digital screens and posters[b] | | | | | |
| | *Organizational level* | | | | | | |

[a] Activities implemented six months after the start of the implementation. [b] Activities implemented 8-10 months after start of the implementation
